# Preparation of Novel Marine *Enterococcus faecium* MSD8 Exopolysaccharide Ointment and In Vivo Evaluation of Its Impact on Cutaneous Wound Healing in Male Albino Rats

**DOI:** 10.1007/s12602-024-10334-z

**Published:** 2024-08-12

**Authors:** Doaa A. Abdel-monem, Soraya A. Sabry, Hanan A. Ghozlan, Eman H. Zaghloul

**Affiliations:** 1 Botany and Microbiology Department, Faculty of Science, Alexandria University, Alexandria, Egypt; 2 National Institute of Oceanography and Fisheries (NIOF), Cairo, Egypt

**Keywords:** Exopolysaccharide, Wound healing, *Enterococcus faecium*, Chemical characterization, Animal study

## Abstract

The current study describes the isolation of exopolysaccharide (EPS) producing lactic acid bacteria (LAB) from marine samples and testing different sugar additives with different proportions for enhanced EPS yield. The isolate MSD8 showed the most potential, yielding 200 mg/L of EPS after being cultivated at 37 °C for 48 h on de Man Rogosa and Sharpe medium (MRS) supplemented with 3% sucrose. The marine isolate MSD8 was identified as *Enterococcus faecium* with 99.58% probability using 16S rRNA gene sequencing. The obtained sequence was deposited in GenBank and assigned the accession number MW924065. The feature of MSD8-EPS was characterized by estimating the total carbohydrate content by UV–vis to be ~ 71%. The FTIR analysis further indicated the presence of characteristic bands of polysaccharide. The cytotoxicity of the produced MSD8-EPS was assessed using human skin fibroblasts (HSF). The IC_50_ was determined to be > 100 μg/mL, which signifies that MSD8-EPS is safe for skin application. The produced EPS was used to prepare a novel ointment, which was tested for wound healing ability in male albino rats. The ointment significantly (P ≤ 0.05) shortened the time needed for wound healing, as it successfully healed the wounds by 94.93% on the 7th day and completely (100%) healed the wound by the 12th day. In comparison, the control group was healed by 73.2% and 84.83%, respectively. The data confirm that the prepared ointment can safely be used for pharmaceutical wound care products.

## Introduction

Lactic acid bacteria (LAB) are a heterogeneous group of bacteria with a broad distribution in nature [1]. They are generally recognized as safe, and various LAB strains can produce beneficial exopolysaccharides (EPS) [2].

Marine LAB strains are adapted to live under harsh environmental conditions. They can be isolated from sponges, seaweeds, and fish in marine environments. Marine LAB and its unique by-products may possess significant value in food processing, fermentation, pharmaceuticals, and biopolymer industries [3]. Moreover, marine LAB-derived EPS serve several functional purposes, including mitigating lactose intolerance, stimulating the immune system, acting as antioxidants, preventing colon cancer, decreasing cholesterol levels, and promoting wound healing [4].

EPSs are complex carbohydrates with a large molecular weight. They are classified as homogeneous or heterogeneous polysaccharides depending on whether they contain a mixture of different monosaccharides or they contain only one type of monosaccharide. Increasing knowledge regarding the impact of EPS from LAB on health with the most recent technologies has led to increasing interest in them due to their major beneficial effectiveness on human health. Several research studies have shown the physical and chemical characteristics and the biological activities of EPS produced by lactobacilli, specifically *Lactobacillus plantarum* [5] and *Bifidobacterium longum* BB-79 [6]. However, little research has been done on the properties of EPS produced by *Enterococcus faecium* from various ecosystems. These studies, however, have mostly focused on biological activities such as antimicrobial, antioxidant, hypocholesterolemia, and antibiofilm action [7].

Skin, as the first immunity barrier, plays a vital function in safeguarding the human body and preserving human health. The skin is colonized by microbiota that maintains its health and homeostasis [8]. Unwanted changes to this balance may happen due to inflammatory conditions, surgical procedures, burns, and accidental wounds [9]. In healthy individuals, wound healing begins immediately after injury. However, in some cases, wounds can take longer or do not heal at all [10]. This slow healing can increase a person’s risk of complications and other infections [11].

Wound healing is a complex process that involves a series of overlapping phases, including hemostasis, inflammation, proliferation, and remodeling [12]. During this process, the skin must balance the breakdown and production of extracellular matrix components, such as collagen, to facilitate the recovery of dermal and epidermal tissues. Production of wound healing drugs is very costly, and many of them may induce side effects. Emerging research has explored using natural polymers, including polysaccharides, as biomaterials for wound healing [13]. EPS produced by marine bacteria, such as *Enterococcus*, have attracted attention for their potential in wound management applications. These microbial-derived polymers exhibit advantageous properties, including high absorption capacity, biocompatibility, and low cost, which make them promising candidates for wound dressings and tissue engineering scaffolds. Compared to synthetic polymers, natural EPS may more closely mimic the extracellular matrix, offering a favorable microenvironment for cell proliferation, migration, and differentiation during wound healing. Further research is needed to fully elucidate how these polymers can enhance wound healing and develop effective formulations and delivery methods for clinical application [14, 15].

Hence, this research aims to isolate and identify novel EPS-producing marine LAB, produce the EPS, and use it to prepare a novel EPS ointment. Moreover, evaluates the prepared ointment efficacy in promoting cutaneous wound healing in albino rats through in vivo assessment.

## Material and Methods

### Isolation of Marine LAB

Various marine samples (Poulet, Pilchard, Gilt-head bream fishes, and shrimps) were obtained from local markets in Alexandria, Egypt. The acquired specimens were transported immediately to the laboratory in an icebox for further processing. In the laboratory, they were dissected, and the gut of each specimen was aseptically transferred to a test tube containing sterile saline and shaken thoroughly. Then, 1 mL of each tube was inoculated in 9 mL de Man Rogosa and Sharpe medium (MRS) broth (Merck, Germany) composed of (g/L) (5: yeast extract, 10: beef extract, 10: peptone, 20: glucose, 2: K_2_HPO_4_, 5: anhydrous sodium acetate, 2: ammonium citrate, 0.58: MgSO_4_⋅7H_2_O, 0.25: MnSO_4_⋅H_2_O, and 1 mL/L Tween 80), and incubated under anaerobic conditions in a candle jar for 48 h at 37 °C for enrichment [16].

After incubation, serial dilutions were prepared for each sample, and 1 mL of each dilution was cultured on sterile MRS agar plates. Then, the Petri dishes were incubated for 48 h at 37 °C in a candle jar under anaerobic conditions. The separate phenotypically different colonies were then picked up and subcultured two or three times on MRS agar plates using the streak plate method for purification. After purification, the obtained isolates were further examined to determine their Gram reaction, morphology, motility, and sporulation. The obtained isolates were tested for catalase production according to [17].

Gram-positive, non-spore-forming, catalase-negative, and non-motile isolates were considered as presumptive LAB, and they were preserved as glycerol stock prepared by mixing 0.5 mL of 70% sterile glycerol with 0.5 mL of the bacterial culture and stored at -4 °C for further study.

### Screening for EPS Production

A screening test was conducted to assess the obtained isolate’s ability to produce EPS. Modified MRS medium supplemented with 10 g/L sucrose was used to cultivate the isolates. The plates were incubated under anaerobic conditions at 37 °C for 48 h. After incubation, the ropiness test was detected by touching the bacterial colonies with a sterile inoculation loop. Mucoid isolates were selected for further study [18, 19].

### Effect of Added Sugars on EPS Production

A preliminary investigation was conducted to assess the influence of different sugars on the yield of the produced EPS. The selected 3 mucoid isolates were cultivated on MRS broth supplemented with different sugars (sucrose, galactose, fructose) with different concentrations (10, 30, and 50 g/L) separately and incubated at 37 °C for 48 h.

After incubation, 1 mL of each tube was centrifuged at 5000 rpm for 15 min to remove bacterial cells. The supernatant was treated with trichloro acetic acid (TCA) (10% w/v) for 30 min to remove proteins, centrifuged at 5000 rpm for 15 min, and pellets were discarded. EPS was precipitated by treating the supernatant with cold absolute ethanol (99%) at 1:3 and storing it at 4 °C for 48 h. EPS was collected by centrifugation for 15 min at 5000 rpm and dried overnight at 37 °C. The dry weight of EPS was calculated as the mean ± standard deviation of three trials [4, 20].

### Mass Production of MSD8-EPS

The isolate with the greatest potential for EPS production (MSD8) was selected for large-scale production of EPS. The chosen isolate was inoculated in MRS broth (1000 mL) with 30 g/L sucrose and incubated at 37 °C for 48 h. The EPS was extracted as mentioned above. Then, the precipitated EPS was partially purified using a dialysis bag with a pore size of 15 KDa (Sigma-Aldrich, USA) immersed in distilled water, and the water was changed twice a day. The partially purified EPS was placed in a drier at 37 °C till complete dryness. The EPS powder was stored in a refrigerator for further studies [21].

### Characterization and Identification of the Selected Isolate

According to the method described by Figueroa-Bossi et al. [22], the chromosomal DNA of the marine isolate MSD8 was prepared by extracting 2 mL of the overnight grown bacterial culture, the bacterial pellets were suspended in a mixture of 500 μL TEN buffer (40 mM Tris-HCl pH 8.0 + 1 mM EDTA pH 8.0 + 150 mM NaCl) then mixed with 25 μL Lysozyme (20 mg/mL) and incubated at 37 ℃ for 2 h. and mixed with 75 μL of 10% SDS. Then, 3 μL of 20 mg/mL of Proteinase K were added, mixed, and incubated at 55 ℃ for 1 h. Up to this step, the mixture became viscous and clear. After adding 100 μL of 5 M NaCl, DNA was extracted with 0.8 mL of phenol/chloroform/isoamyl alcohol (24:24:1) and centrifuged for 10 min at 13,000 rpm. The aqueous phase was transferred into a new clean tube, and DNA was precipitated using 0.8 mL of chloroform. After centrifugation for 10 min at 13,000 rpm, the DNA in the aqueous layer was precipitated with 0.7 mL of Isopropanol and re-centrifuged for 10 min at 13,000 rpm. The pellet was then washed with 250 μL of 70% Ethanol and dried.

The DNA pellet was dissolved in 50 μL of TE buffer, which consisted of 10 mM Tris–HCl at pH 8.0 and 1 mM EDTA at pH 8.0. The integrity of the isolated DNA was verified using gel electrophoresis before the polymerase chain reaction (PCR).

#### 16S rRNA Sequence Analysis

The amplification of the 16S rRNA gene was conducted using universal primers 27F/1492R, with the sequences 27F 5′-AGAGTTTGATCCTGGCTCAG-3′ and 1492R 5′-TACGGYTACCTTGTTACGACTT-3′. The PCR amplification and product sequencing were conducted by Sigma Scientific Services Company at the GIS Research Centre in Cairo, Egypt. The PCR amplicons were subjected to automated sequencing utilizing the BigDye chain termination approach [23].

The acquired sequence was analyzed using the BLAST program offered by the NCBI, and the phylogenetic analysis was conducted utilizing the resources supplied by the website https://www.phylogeny.fr.

### Assessment of Hemolytic Activity

To further explore the safety of isolate MSD8, it was streaked over blood agar plates containing 5% (w/v) sheep blood and incubated at 37 °C for 24 h. Following the incubation period, the plates were inspected for indications of hemolysis [24].

## Characterization of MSD8-EPS

### Total Carbohydrate Content (%)

The phenol–sulfuric method, as described by Albalasmeh et al. [25], was used to assess the total carbohydrate content of MSD8-EPS, with glucose used as standard. The standard solution was prepared by dissolving 100 mg of glucose in 100 mL of distilled water. The EPS powder (10 mg) was dissolved in 100 mL distilled water. A series of test tubes were used to prepare the standard solution, with volumes ranging from 0 to 1 mL in increments of 0.2 mL. The volume was made up to 1 mL by adding distilled water. One volume of phenol was added to each tube, followed by 5 mL of 96% sulfuric acid and shaking. After 10 min, the tubes were placed in a water bath and incubated at 25–30 °C for 20 min. The standard and sample absorbances were measured at 490 nm. The sugar content was determined by using the calibration curve and applying the following equation:

$${\varvec{Glucose \; concentration}}\;\left({\varvec{m}}{\varvec{g}} \boldsymbol{\%}\right)=\;{\varvec{Absorbance \; of \; sample}} \;* \; \varvec{the \; interaction\; factor}$$

### Fourier-Transform Infrared Spectroscopy (FTIR) Analysis

The MSD8-EPS, weighing around 2.0–3.0 mg, underwent FTIR analysis to determine its distinctive functional groups. This analysis was conducted utilizing a Bruker spectrometer coupled with a horizontal attenuated total reflection (ATR) system (ALPHA, Germany) at Nawah Scientific Inc. (Mokatam, Cairo, Egypt). The MSD8-EPS powder was mixed with KBr and then pressed into a disk. The IR spectrum was acquired between the 4000 – 400 cm^−1^ frequency ranges under ambient conditions [20].

### Determination of MSD8-EPS Cytotoxicity

In order to ascertain the safety of MSD8-EPS for use in wound healing applications, its cytotoxicity towards human skin fibroblasts (HSF) was assessed at Nawah Scientific Inc. (Mokatam, Cairo, Egypt). HSF was kept at 37 °C in a humidified 5% (v/v) CO_2_ environment enriched with 100 units/mL penicillin, 100 mg/mL streptomycin, and 10% of heat-inactivated fetal bovine serum in Dulbecco's Modified Eagle's Medium (DMEM). The Sulforhodamine B (SRB) test was used to measure the vitality of the cells [26]. 96-well plates were filled with aliquots of 100 μL cell suspension (5 × 10^3^ cells), and the plates were incubated for 24 h in a complete medium.

For 72 h, the cells were exposed to a second aliquot of 100 μL media that included MSD8-EPS at different concentrations (0.01, 0.1, 1, 10, and 100 μg/mL). After that, the medium was changed to 150 μL of 10% TCA to fix the cells, and they were incubated for 1 h at 4 °C. After five rounds of distilled water washing, the cells were suspended in 70 μL of SRB (0.4% w/v) and stored at ambient temperature for 10 min in the dark. After washing with 1.0% acetic acid, the plates were left to air dry. Subsequently, the protein-bound SRB stain was dissolved by adding 150 μL (10 mM) of TRIS-hydrochloride solution. The absorbance was determined at 540 nm using an Omega microplate reader (Ortenberg, Germany). The product is considered safe if IC_50_ is used at a concentration greater than 100 (IC_50_ > 100).

$$The\; \% \; viability\; was\; calculated\; as\; follows\!\!: (\frac{A\; sample - A\; blank}{A\; control - A\; blank})*100$$

### Preparation of MSD8-EPS ointment

The possibility of using the produced EPS for wound healing was examined on experimental animals, and for this purpose, it was formulated as an ointment. The ointment was prepared by dissolving 15 mg/mL of the EPS in a sterile solution of 30% glycerol and mixing until the ointment was formed [27].

### In Vivo Cutaneous Wound Healing Ability Evaluation

The ability of the produced MSD8-EPS for wound healing was evaluated according to Ntenis et al. [28] with modification. Briefly, a group of 20 male albino rats weighing 150–200 g was selected. The rats were housed under controlled conditions (12 h light–dark cycle; 20 ± 3 °C; 60–70% humidity) in plastic cages with coverlids made of stainless steel and fed with a specific rodent pellet diet and water [29, 30]. The rats were kept at the animal house unit of the Medical Technology Center, Alexandria University, Egypt. The procedure used in this research underwent a thorough evaluation and received approval from the Institutional Animal Care and Use Committee at Alexandria University (IACUC) with code **(AU 04210617101)** before the initiation of this experiment.

A Mixture of xylazine (10 mg/kg) and ketamine (90 mg/kg) (Ranglab, Australia) was used to anesthetize the experimental rats. Once the rats had lost consciousness, the fur on the dorsal side was aseptically shaved with a surgical blade disinfected with 70% alcohol, and the predicted area of the wound was marked on the shaved skin. The wound was inflicted with sections 1–1.5 cm with the aid of 70% alcohol-disinfected forceps and surgical scissors. After cleaning the wounds with cotton immersed in alcohol and betadine, the rats in each of the experimental groups received the respective topical application.

The Experimental Rats Were Categorized Into 4 Distinct Groups:

- Group I served as a negative control and was not treated, and the wound was cleaned with saline.
- Group II was treated with MSD8-EPS ointment (200 µL).
- Group III was treated with Candex cream (from AIM pharmaceutical company) and served as standard reference (positive control).
- Group IV was treated with 15 mg/mL glycerol (Control), the same concentration used to prepare the ointment.

The wounds were covered with surgical dressings. The rats were housed individually, and the topical application was applied day after day till wound healing occurred.

### Wound Contraction Measurements

Digital photos of each rat's wounds were taken on days 0, 3, 5, 7, 9, and 12. Subsequently, the healing activity was evaluated according to Krishna et al. [30] as the percentage of wound contraction, which was determined by measuring the wound size using a caliper. The contraction percentage was calculated by subtracting the original and final wound sizes for each rat.

The percentage of wound healing was calculated as follows:

$${Wound\; Contraction}_{(\%)}=\frac{Initial\; area\; of\; the\; wound-Specific\; day\; wound\; size}{initial\; area\; of\; wound}*100$$

### Statistical Analysis

All findings were reported as mean value ± SD, and the Post Hoc test (Tukey) for pairwise comparisons was applied to test statistically significant differences between more than two groups [31]. A probability value of ≤ 0.05 was considered significant. The statistical analyses were carried out using the SPSS version 20.0 software package **(**Armonk, NY: IBM Corp**)**.

## Results and Discussion

### Isolation of EPS-Producing Marine LAB

A total of 16 bacterial isolates were obtained on MRS agar plates from different marine samples used. Data in Table 1 show some of the important phenotypic characters of the isolates. Sixteen isolates were obtained; only 14 were confirmed as Gram-positive cocci or rods, catalase-negative, non-spore-formers, and they were considered as presumptive LAB. Two isolates, MSD1 and MSD6, were excluded because they were Gram-negative or catalase-positive, respectively [32].

**Table 1:** Phenotypic characterization of the bacterial isolates obtained on MRS agar plates

**Isolate**	**Source of isolation**	**Shape**	**Gram staining**	**Catalase reaction**	**Mucoid appearance**
**MSD1**	Poulet fish gut	Cocci	-ve	-ve	-
**MSD2**	Pilchard gut	Cocci	+ve	-ve	+
**MSD3**	Pilchard gut	Cocci	+ve	-ve	+
**MSD4**	Gilt-head bream gut	Cocci	+ve	-ve	+
**MSD5**	Gilt-head bream gut	Cocci	+ve	-ve	+
**MSD6**	Gilt-head bream gut	Rods	+ve	+ ve	-
**MSD7**	Shrimp gut	Rods	+ve	-ve	+
**MSD8**	Shrimp gut	Cocci	+ve	-ve	++
**MSD9**	Shrimp gut	Rods	+ve	-ve	+
**MSD10**	Shrimp gut	Rods	+ve	-ve	++
**MSD11**	Shrimp gut	Rods	+ve	-ve	+
**MSD12**	Shrimp gut	Rods	+ve	-ve	++
**MSD13**	Shrimp gut	Cocci	+ve	-ve	+
**MSD14**	Shrimp gut	Rods	+ve	-ve	+
**MSD15**	Shrimp gut	Rods	+ve	-ve	+
**MSD16**	Shrimp gut	Cocci	+ve	-ve	+

-ve negative, +ve positive, – not mucoid, + mucoid, ++ highly mucoid

### Effect of Added Sugars on EPS Production

**Fig. 1:**
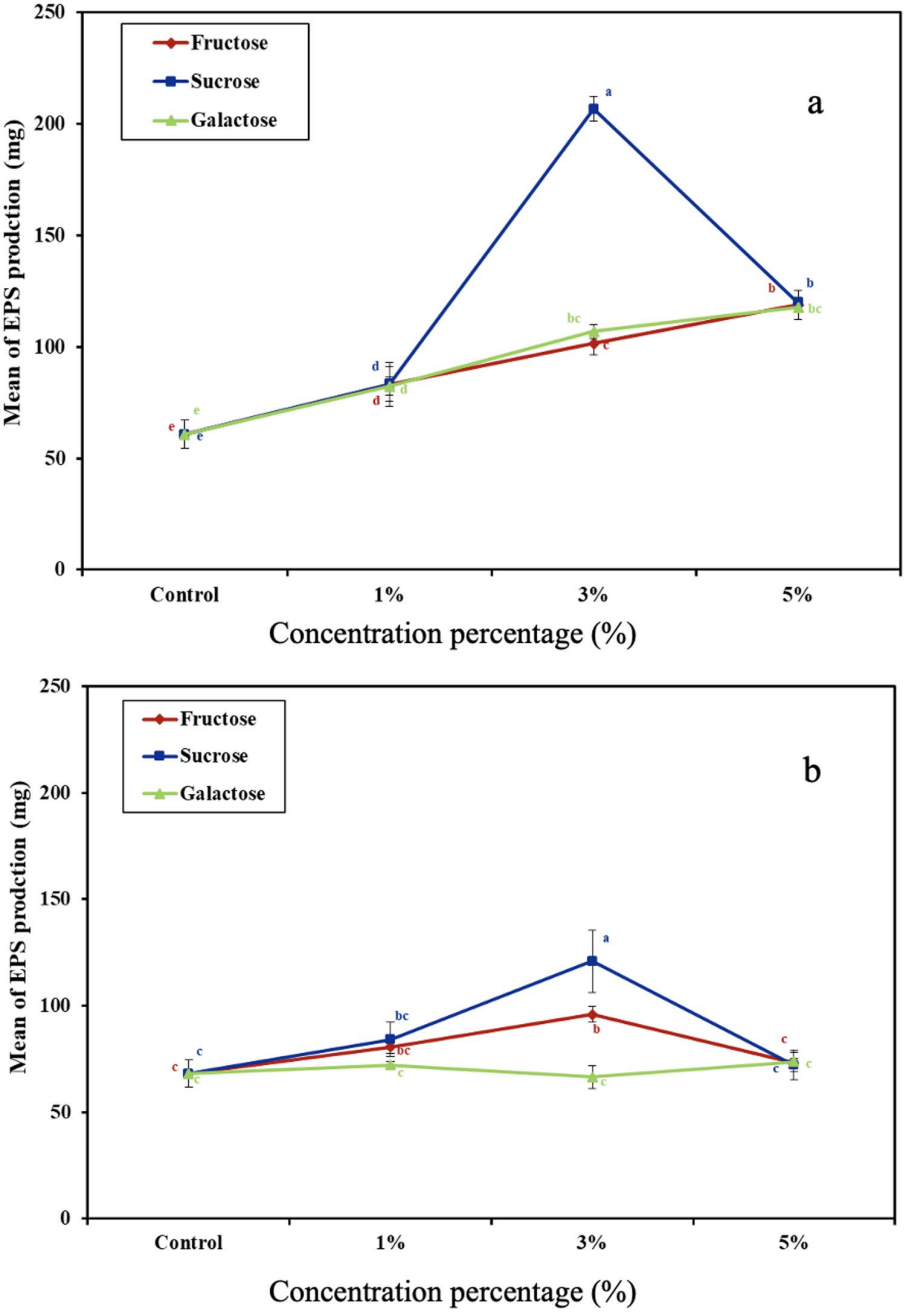
EPS production yield by MSD 8 (**a**) and MSD 12 (**b**) grown at 37 °C for 48 h in MRS medium enriched with different sugars at different proportions; MRS with no added sugars used as control

The most promising mucoid isolates, MSD8 and MSD12, were further examined for EPS production yield in the presence of sucrose, fructose, or galactose. Data in Fig. 1 show that isolate MSD8 significantly (P ≤ 0.05) produced a higher EPS yield (200 mg/L) in media supplemented with sucrose (30 mg/L) in comparison to the EPS yield produced by MSD12 (121 mg/L) and greater than the yield of EPS produced in the presence of galactose or fructose.

The isolation of EPS-producing LAB from marine samples was previously documented; for example, the EPS-producing *Enterococcus faecium* MC13 was isolated from fish gut [33], and *Lactiplantibacillus plantarum* EI6 was isolated from marine shrimp. It was documented as a promising EPS producer [4].

On the other hand, the lowest EPS production yield was obtained in the case of MRS with no sugar additives, which recorded 60 and 68 mg/L for isolate MSD8 and MSD12, respectively. The yield of EPS (200 mg/L) from the presumptive LAB isolate MSD8 in the presence of sucrose (30 mg/L) is higher than that reported by Sørensen et al. [34], for *Staphylococcus thermophilus* (108 mg/L). Since isolate MSD8 had the maximum EPS yield, it was chosen for further investigations.

According to Abdulhamid et al. [35], every bacterial strain develops distinctive EPS with unique structures and biological activities. Additionally, microbial EPS may be used alone or in conjunction with other substances for diverse applications in the pharmaceutical and biomedical sectors. Furthermore, the amount, chemical structure, and bioactivity of the derived EPS may be influenced by the producing strain, culture conditions, and the composition of the culture medium [36]. Thus, for the synthesis of new EPS with notable biological activities, careful strain selection, optimization of production conditions, and the functional groups present in the EPS structure play a crucial role in evaluating new EPS with notable biological activity [37].

The screening for LAB capable of EPS production by the slimy mucoid appearance of the colony on solid media has been adopted for many years in many studies as a preselection approach. This approach is advantageous as it helps in the rapid and cost-efficient screening of multiple strains. However, this approach is very hard to standardize as the colony morphological distinctions are identified by the researcher’s subjective assessment. Furthermore, some LAB EPS may not have substantial slime-forming capabilities and may be mistaken as false negatives [38]. One way to overcome this experimental challenge proposed by Rühmann et al. [39] is to compare different colony properties between induced and non-induced production circumstances. The carbon source and cultivation conditions significantly affect the colony's size, shape, and appearance, as well as the yield of EPS production [40]. Therefore, cultivating LAB strains on media supplemented with different carbon sources can be used to screen high EPS-producing LAB effectively [38].

The EPS yield recorded by Rahnama Vosough et al. [41] was 2.39 and 2.68 g/L for *E. faecium* T52 and *E. faecium* R114, respectively, in an MRS medium supplemented with 40% sucrose [41]. This yield is much greater than the yield obtained in this study. The lower production efficiency of EPS from MSD8 may be connected to the culture medium's ingredients and the TCA used to purify the EPS. The yield of EPS is further decreased by using TCA in EPS purification because a significant percentage of EPS is precipitated with TCA and eliminated along with proteins [42]. According to the findings of other studies, the carbon source substantially impacts the production of EPS [41], and the culture medium utilized in our investigation contains 3% sucrose. For instance, in the work conducted by Kanmani et al. [33], *E. faecium* MC13 produced more EPS when grown in a medium that included sucrose.

### Molecular Identification of MSD8

**Fig. 2:**
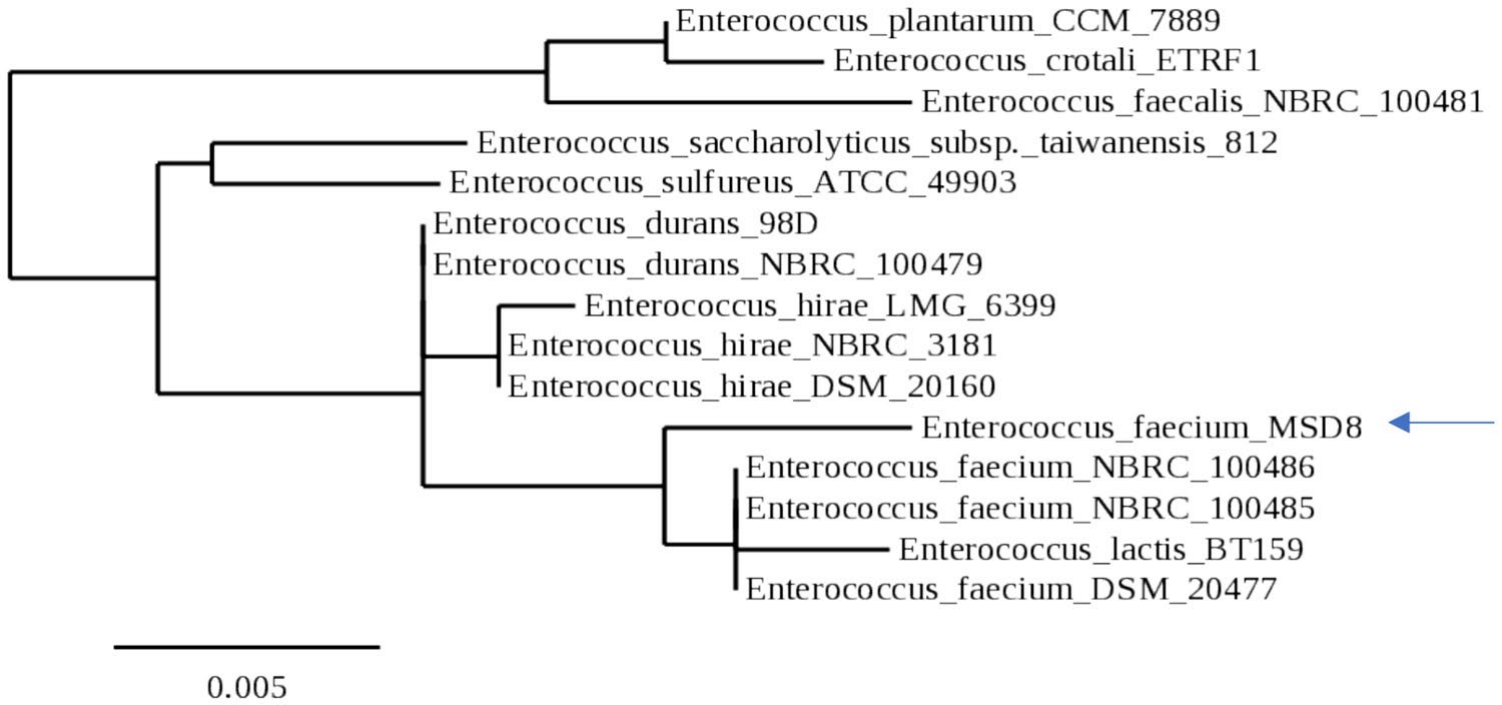
Isolate MSD8 16S rRNA based dendrogram showing the phylogenic position of isolate MSD8 among representatives of related bacterial species

The marine isolate MSD8 was identified via 16S rRNA gene sequencing. It was classified as *Enterococcus faecium* with a similarity of 99.58%. The resulting sequence was deposited in GenBank and assigned the accession number MW924065. Figure 2 illustrates the evolutionary correlation between MSD8 and its closely related counterparts in the NCBI database. It belongs to the taxonomic classification of Kingdom: Bacteria, Phylum: Bacillota, Class: Bacilli, Order: Lactobacillales, Family: Enterococcaceae.

The isolation of *E. faecium* from marine samples was previously reported by Abuohashish et al. [43]. Moreover, Kanmani et al. [33] isolated the EPS-producing strain *E. faecium* MC13 from the fish gut.

### Hemolytic Activity of Isolate MSD8

Hemolysis is regarded as a significant virulence factor in pathogenic bacteria. Therefore, this study tested the growth of the marine isolate *E. faecium* MSD8 on blood agar. MSD8 exhibited no signs of hemolysis (γ-hemolytic), suggesting that MSD8 is safe and appropriate for use in biotechnological and industrial contexts [4].

## Characterization of MSD8-EPS

### Total Carbohydrate Content (%)

Unlike *L. plantarum*, limited studies have been undertaken for the characteristics of EPS produced by *E. faecium* isolated from fresh and processed fish products [44].

It was reported that EPS extracted from *E. faecium* has great roles in our lives, as it was realized by Ayyash et al. [44] that the EPS produced from *E. faecium* MS79 has a remarkable activity as an antidiabetic agent by inhibiting α-amylase and α-glucosidase; also, it exhibits antiproliferative effects on colon and breast cancer cell lines.

Therefore, the marine isolate *E. faecium* MSD8 was selected for mass EPS production, and the MSD8-EPS was characterized by determining total carbohydrate contents. The carbohydrate quantity is crucial not only for indicating the purity of the produced EPS but also for determining the functional characteristics of the EPS and specifying its potential applications [4]. The study determined the carbohydrate contents in the MSD8-EPS by measuring the reducing sugar, which accounted for about 71%. The sugar concentration exceeded the published value of Kaur et al. [45] for *Alcaligenes faecalis* B14 (63%) but fell short of the carbohydrate content of the EPS from *Rhodobacter johrii* CDR-SL 7Cii (86.82%) as reported by Sran et al. [46].

### FTIR Analysis

**Fig. 3:**
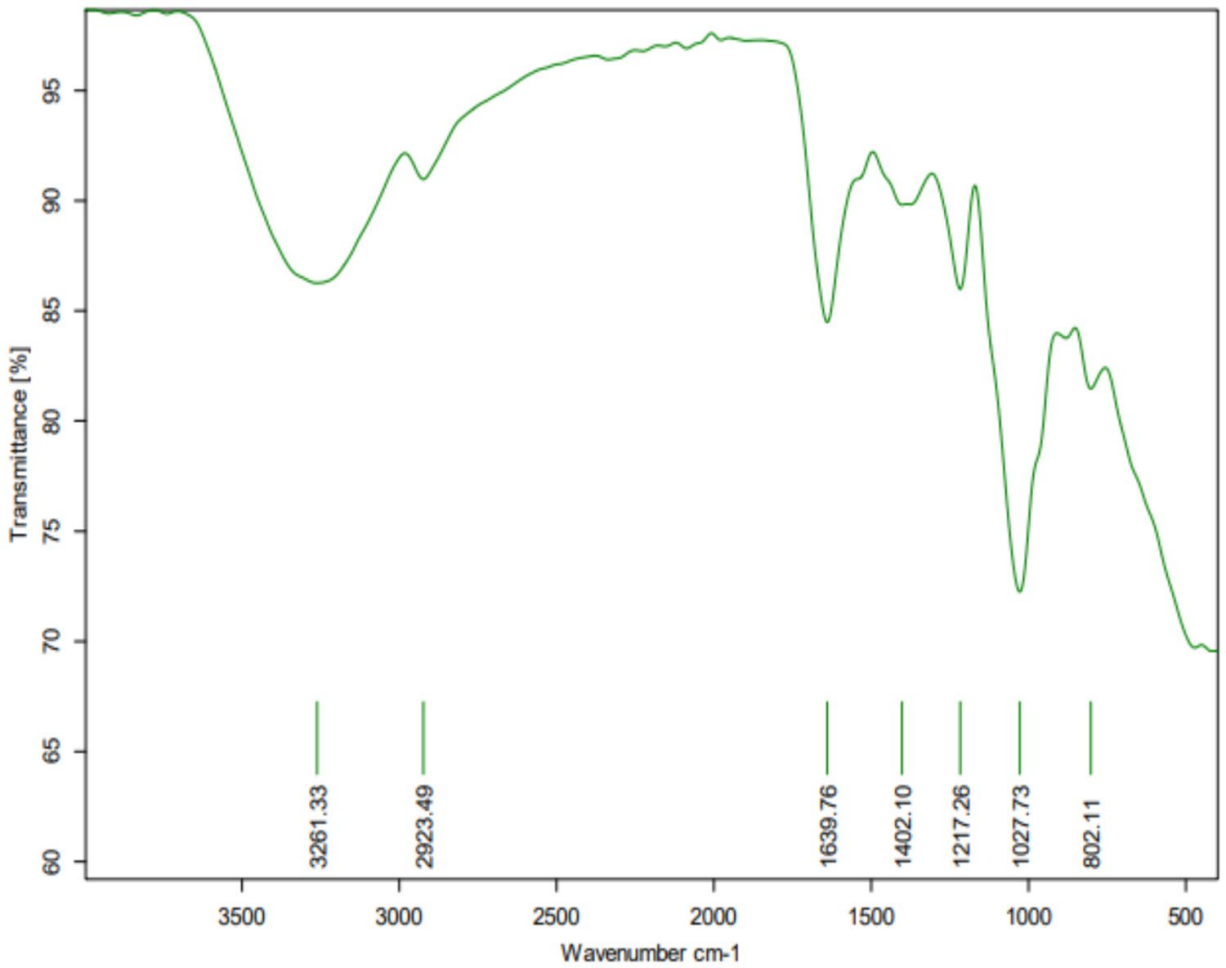
FTIR spectrum of partially purified polysaccharides isolated from marine *E. faecium* MSD8

The FTIR spectrum was used to investigate the major functional groups of the produced MSD8-EPS as it recorded characteristic bands and peaks of polysaccharides at absorbance mode from 4000–400 cm^−1^ [47]. The IR bands were primarily determined based on the prior published spectra of polysaccharides (16). The FTIR analysis showed clear absorption peaks at 3261.33, 2923.49, 1639.7, 1402, 1217, 1027, and 802 cm^−1^ (Fig. 3). The broad absorption peak within the range 3200–3300 cm^−1^ is attributed to the stretching vibration of intensive hydroxyl groups (O-H); it might be included in intra-and inter-molecular linkage in the EPS chains [33, 48].

The absorption peak at 2924 cm^−1^ corresponds to the stretching vibration of the C-H bonds in the methyl or methylene groups found in hexoses (such as galactose and glucose) and deoxyhexoses (such as rhamnose and fructose), which is a distinctive feature of polysaccharides [49]. The two peaks, 3261.33 and 2923.49, are considered strong evidence of polysaccharides' existence [50]. The band at 1639.7 cm^−1^ is attributed to the stretching vibration of the carboxyl group (C=O). In addition, the absorption peak at around 1639 cm^−1^ is documented to be caused by α-glycosidic bonds and hydrogen bond absorptions associated with trace water [51]. The peak obtained at 1402 cm^−1^ shows the presence of C=C in the aromatic group [52]. The peak near 1200 cm^−1^ (1217 cm^−1^) indicates polysaccharides contain α-pyranose. Indeed, the carbohydrates show high absorbency during this region, which is the so-called fingerprint region, where the bands' position and intensity are specific for each polysaccharide, allowing the chance of identification [53]. The signal at 1027 cm^−1^ correlates with the C–OH vibration [54]. Along with these peaks, one of the most documented characteristic peaks at the 802 cm^–1^ region was also detected, indicating that the MSD8-EPS contained β-type glycosidic linkages between sugar monomers [53].

### MSD8-EPS Cytotoxicity Evaluation

**Fig. 4:**
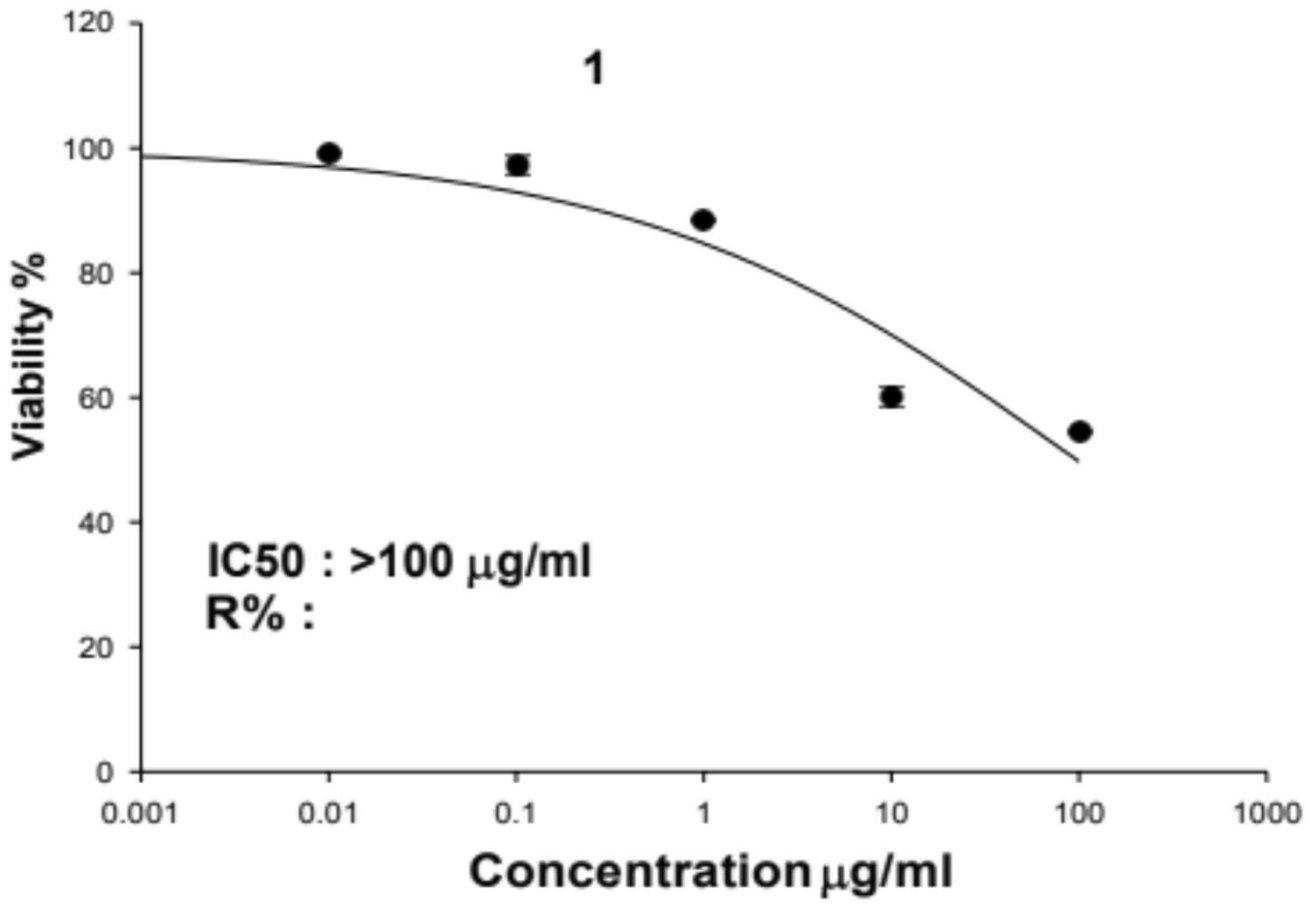
The viability percentage of cells treated with different concentrations of *E. faecium* MSD8 exopolysaccharide for 72 h

To determine its safety, the cytotoxicity of MSD8-EPS was evaluated against HDF. Figure 4 shows the percentage of viable cells after treatment with different concentrations (0.01, 0.1, 1, 10, 100 μg/mL) of MSD8-EPS.

The highest inhibitory effect on the proliferation of cells was observed at 100 mg/mL after 72 h of treatment with the EPS at a statistically significant level (P ≤ 0.05). However, at concentrations of 0.01 μg/mL, 0.1 μg/mL, and 1 μg/mL, there was only a slight reduction in cell viability. Whereas at the concentrations of 0.01 μg/mL and 0.1 μg/mL of EPS, no cytotoxic effects were observed on HDF cell lines. The IC_50_ value, indicating the concentration at which a substance inhibits 50% of cell viability, was more than 100 μg/mL. This indicates that even at doses as high as 100 μg/mL, MSD8-EPS did not significantly diminish the viability of skin cells, indicating its safety.

### In Vivo Cutaneous Wound Healing Ability Evaluation

Wounds are physical injuries that cause a gap or damage in the skin. Common wound symptoms often encompass bleeding, diminished feeling or function below the wound site, localized heat and redness, discomfort, and swelling of tissue in the affected area [55], and perhaps the formation of pus cells. Adequate wound healing is crucial for restoring disrupted skin [56]. An optimal wound-healing compound should meet diverse requirements while enabling smooth and scar-free rapid healing.

**Fig. 5:**
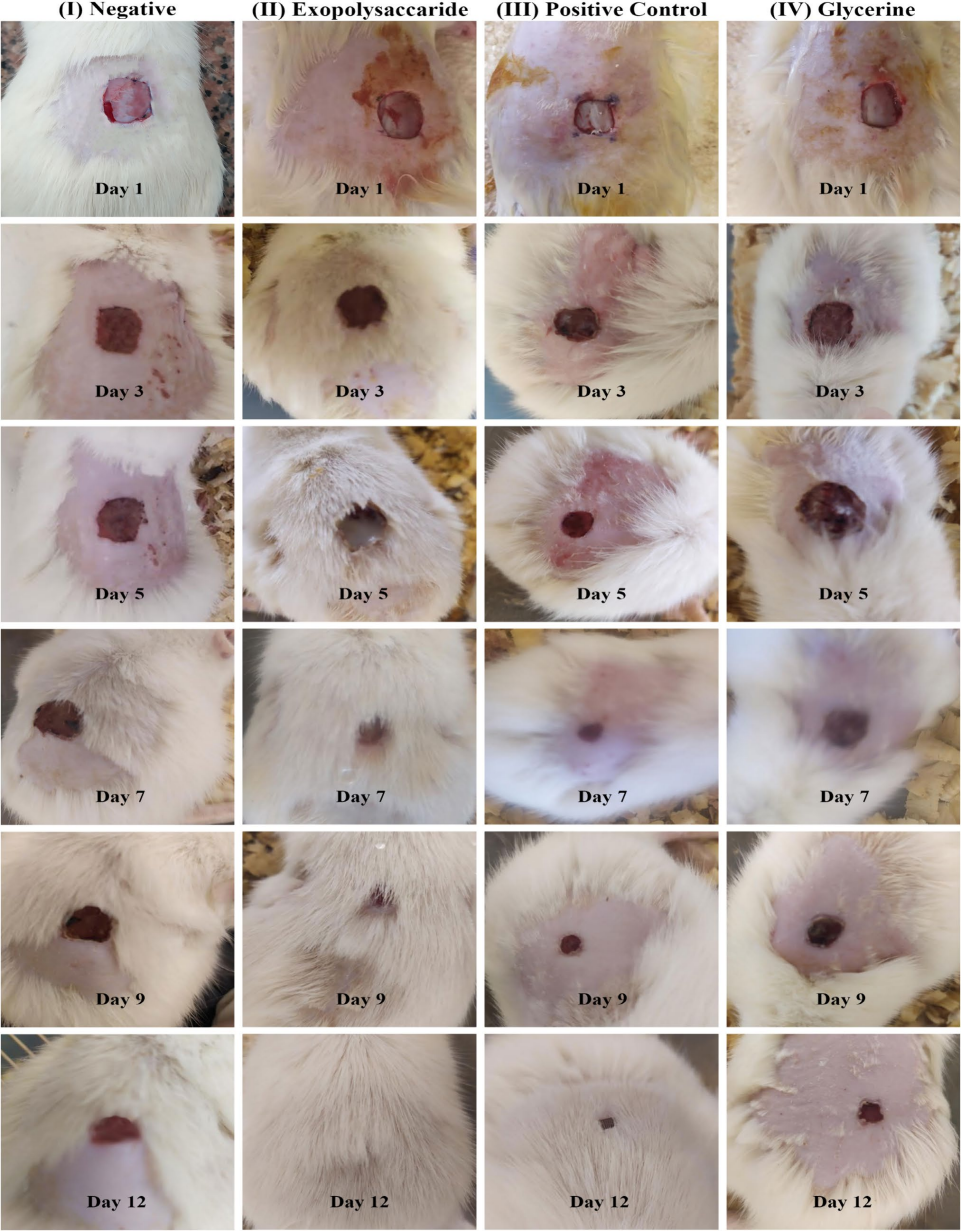
Photographic representation of the wound contraction rate in male albino rat groups for 12 days. I: Negative control group, II: MSD8-EPS treated group, III: Positive control group, and IV: Glycerine group

Therefore, the wound healing efficacy of MSD8-EPS was evaluated based on the visual appearance and quantitative assessments of the injured region in the 4 groups of rats for 12 days, as illustrated in Fig. 5.

**Fig. 6:**
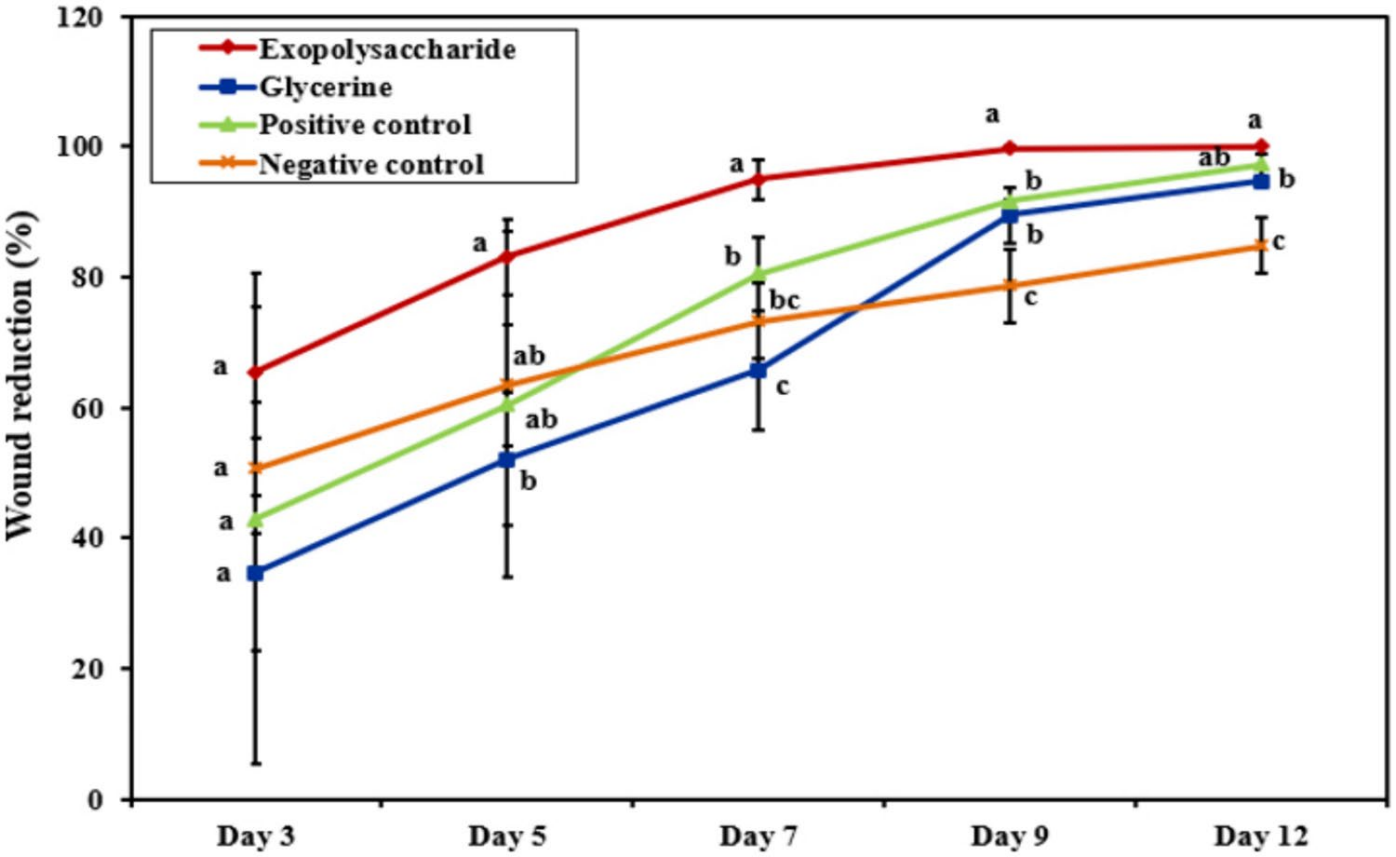
The rate of wound contraction in the negative control, Positive control, Glycerine, and MSD8-EPS treated groups for 12 days. Values are expressed as mean ± SD; (P ≤ 0.05)

On the first day of the experiment, the wound of each rat of the 4 groups showed the same bright red color, displaying the blood on the underlying muscle after the skin removal. On the 3rd day, brown color scabs were observed in all groups and the MSD8-EPS treated group (Group II) with a decrease in the wound area (Fig. 6), which is believed to be evidence of initiating the healing process through the formation of blood clots containing cellular debris [27]. On the 5th day of the experiment, one of the rats in groups I and III died, and the scab tissue of (Group II) and (Group IV) was dethatched. The wounds became healthy, clear, and pinkish, while the scab persisted in (Group I), and (Group III), even after the 9th day of the healing process. On the 7th day, the wounds treated with MSD8-EPS exhibited increased homogeneity and consistency in texture, showing a significant (P ≤ 0.05) wound closure compared to the rest of the groups. On the 9th day of the experiment, almost complete closure of wounds of rats treated with MSD8-EPS (Group II) was observed. After 12 days, the scab of all rats fell, and complete wound healing was observed in (Group II), reflecting the wound repairing and regeneration of the normal tissue.

The data indicated a significant (P ≤ 0.05) wound healing activity in rats treated with MSD8-EPS compared with other groups (I, III, and IV).

The wound closure percentage in the rats treated with MSD8-EPS was 94.93% on the 7th day and 100% by day 12. In the negative control (group I), 73.2% and 84.83% wound closure percentages were observed, respectively. Groups III and IV showed relative measurements in the 9th and 12th days of wound healing as the topical application of Glycerine and “Candex” increased regularly and similarly. The wound contraction percentage decreased the likelihood of infection and expedited epithelization and cell proliferation compared to the wounds of Group I, which exhibited a much-delayed healing process.

According to the obtained data, the produced MSD8-EPS ointment accelerates the wound healing phases, which are (a) clotting and coagulation (hemostasis), (b) inflammation, (c) granulation, and finally (d) remodeling [57] and healing normally occur in a few days, which indicate that MSD8-EPS is a natural, safe, and biocompatible polymer promising for the wound care industry [58].

Microbial EPS, along with other naturally occurring polysaccharides, have attracted considerable interest in recent years due to their potential to modulate the inflammatory response in wound healing. An example of this is the utilization of Glucan in the therapy of wounds, which has been scientifically demonstrated to augment the number of macrophages and improve the processes of fibroplasia, re-epithelialization, and wound strength. Furthermore, Xin et al. [59] showed that administering water-soluble yeast (WSY) glucan to macrophages resulted in a notable and proportional increase in the production of inflammatory mediators. Furthermore, the administration of WSY glucan induced alterations in the morphology of macrophages, enhancing their phagocytic capacity and facilitating wound healing [60].

To the best of our knowledge, no prior information is available about the wound-healing properties of EPS produced by marine *E. faecium*. However, the present results are in accordance with prior research on the wound-healing capacity of EPS produced by LAB, as Trabelsi et al. [27] reported the wound-healing properties of EPS produced by *Lactobacillus* sp. Ca6 after 14 days. Moreover, it was documented by Salimi and Farrokh [61] that the extracted EPS from *E. faecium* MS79 showed promising antibacterial activity, which may help prevent wound area infection and accelerate wound healing. Similar results were presented by Zaghloul et al. [62], who documented a significant antimicrobial activity of EPS produced by marine *Enterococcus* sp., which may explain the promising wound healing activity of *E. faecium* derived EPS.

## Conclusion

The current study describes the isolation of EPS-producing LAB from marine samples and tests different sugar additions with varying concentrations to increase EPS yield. MSD8, the most promising isolate, produced 200 mg/L EPS in MRS medium supplemented with 3% sucrose after 48 h at 37 °C. The marine strain MSD8 was molecularly identified as *Enterococcus faecium* with a 99.58% probability. Moreover, the MSD8-EPS was prepared and preliminarily characterized as having a total carbohydrate content of ~ 71%, estimated by UV vis. The FTIR examination revealed characteristic polysaccharide bands. The cytotoxicity of MSD8-EPS was further evaluated against HSF and showed IC_50_ > 100 μg/mL. A novel EPS-based ointment was prepared and evaluated for wound healing in male albino rats. The ointment successfully cured the wounds by 94.93% on the 7th day and 100% on the 12th day, reducing wound healing time compared to the control, which indicates the suitability and safety of the prepared ointment for pharmaceutical wound care products.

## Data Availability

The datasets generated and/or analyzed during the current study are available in the GenBank and assigned the accession number MW924065.
